# Surgical stress and postoperative complications related to regional and radical mastectomy in dogs

**DOI:** 10.1186/s13028-015-0121-3

**Published:** 2015-06-24

**Authors:** Rodrigo S. Horta, Mariana S. Figueiredo, Gleidice E. Lavalle, Mariana P. Costa, Rubia M. C. Cunha, Roberto B. Araújo

**Affiliations:** Veterinary School, Universidade Federal de Minas Gerais - UFMG, Avenida Antônio Carlos, 6627, caixa postal 567, CEP 30161-970 Belo Horizonte, Brazil

**Keywords:** Dogs, Mammary neoplasms, Surgery, Pain, Regional mastectomy, Unilateral radical mastectomy

## Abstract

**Background:**

Surgery is the treatment of choice for regional control of mammary neoplasms in female dogs. Various surgical techniques may be used, as long as mammary gland anatomy, lymphatic drainage, and known prognostic factors are respected. The purpose of this study was to compare surgical stress—including duration of surgery, nociception and hematological changes—and postoperative complications in dogs undergoing regional and unilateral radical mastectomy. Eighteen dogs were selected for each technique. Postoperative pain (nociception), hematological changes, and postoperative complications were compared between the two groups.

**Results:**

The group treated with radical mastectomy had a longer surgical duration, showed more intense physiological changes, achieved higher scores on nociception scales, and experienced more postoperative complications.

**Conclusion:**

Compared to regional mastectomy, radical mastectomy was associated with longer surgical duration, greater nociceptive stimulus, greater surgical stress, and higher incidence of postoperative complications in dogs. Although evaluation of long-term results was not a goal of this study, it is suggested that postoperative recovery and patient quality of life should be considered when choosing a surgical approach for treating mammary tumors in dogs.

## Background

Mammary gland tumors represent 42 % of all tumors in female dogs [1]. Despite many efforts to develop new treatment modalities, surgery remains the treatment of choice for most dogs with mammary tumors [1, 2]. Different surgical techniques may be applied to promote local control of disease. The simplest and least invasive procedure necessary to remove all nodules and adjacent lymphatic tissue should be selected [1]. In a prospective study, MacEwen et al. [3] did not find differences in overall survival time and relapse rate when comparing simple mastectomy and radical mastectomy in 144 dogs. Nevertheless, some researchers have suggested performing aggressive procedures as a prophylactic measure owing to the possibility of development of new lesions in the remaining mammary tissue [4, 5]. Advantages and disadvantages of each surgical procedure have been thoroughly debated [6]; however, to our knowledge, postoperative aspects have not been considered.

Although mastectomy is associated with a relative low morbidity, it is considered an invasive surgery that may cause moderate to severe pain. Postoperative complications are common, especially when more aggressive surgical techniques are used [7, 8].

Aggressive procedures with extensive incisions and resections probably are associated with higher nociception and postoperative stress, compromising patient quality of life. Those often are performed as prophylactic surgeries without an adequate clinical indication or evident benefits to the patient [1, 2].

The purpose of this study was to compare surgical stress (including duration of surgery, nociception and hematological changes) and postoperative complications in dogs undergoing regional and unilateral radical mastectomy.

## Methods

### Animal selection

Thirty-six female dogs of different breeds with a clinical diagnosis of mammary tumor were subjected to regional mastectomy (18 dogs) or unilateral radical mastectomy (18 dogs). Patients were excluded if they presented abnormalities, such as anemia, azotemia, coagulation disorders, severe cardiac impairment, and pulmonary metastasis visible on thoracic radiographs, during preoperative examinations. Patients with solitary lesions were not included, as they were subjected to nodulectomy, whenever lesion was smaller than 1 cm, or to simple mastectomy, if the lesion size ranged through 1–3 cm.

The Ethics Committee on Animal Experimentation of Universidade Federal de Minas Gerais, Brazil, approved this study (protocol number 23/2011) and the authorization for inclusion of each patient was obtained from its respective owner with a free and informed consent form.

### Surgical technique selection

Lesions on each mammary gland were identified properly and characterized according to size, adherences, necrosis, and ulceration. Clinical staging (three-view thoracic radiographs and abdominal ultrasound) was performed for each patient. When multiple nodules were present, the largest lesion was considered for staging.

Regional mastectomy was indicated for removal of axillary or inguinal mammary lymphatic connections when a single lesion was larger than 3 cm. Cranial abdominal mammary gland was occasionally removed when performing regional mastectomy to achieve clean surgical margins or when lesions of 1–3 cm also were located in this gland. Radical mastectomy was performed when lesions > 3 cm were located on cranial abdominal gland. Regional and radical mastectomies also were performed despite size of lesions when disperse nodules were present along the mammary tissue, in order to create one single surgical wound. An elliptical incision was made around the mammary glands to be excised and subcutaneous tissue was dissected until exposure of muscle fascia. Major blood vessels were ligated, with a 2-0 or 3-0 poliglecaprone suture, including superficial epigastric vessels, whenever inguinal mammary gland was removed. Poliglecaprone also was used to advance the skin toward the center of the defect with a walking suture (2-0 or 0) and a subcuticular continous suture (3-0). Simple interrupted sutures were used to appose skin with a 3-0 monofilament nailon. Surgical duration was defined as the moment from surgical incision to conclusion of skin suture. Surgical wound was measured after conclusion of procedure, in centimeters.

Antibiotic treatment was performed with sodium cephalothin^1^ (30 mg/kg intravenously [IV]) 30 min before and after surgery and every 8 h during hospitalization. After discharge, patients were treated with cephalexin^2^ (30 mg/kg orally [VO]) every 12 h until 10 day after surgery.

All dogs were premedicated with acepromazine maleate^3^ (0.05 mg/kg intramuscularly [IM]) and morphine sulphate^4^ (0.3 mg/kg, IM). Anesthetic induction was performed with propofol^5^ (5 mg/kg, IV). After orotracheal intubation, anesthesia was maintained with isoflurane^6^ in an inhalant anesthesia machine with vaporizer and a semiclosed circuit for dogs larger than 7 kg, and with a Rees-Baraka system for dogs smaller than 7 kg.

Immediately after surgery, each animal was medicated with morphine sulphate (0.2 mg/kg, IV) and meloxicam^7^ (0.2 mg/kg, IV). In the first 24 h, animals were kept in the Veterinary Hospital, with compression bandages. Analgesia was performed with tramadol chlorhydrate^8^ (3 mg/kg, IV) 6 h after surgery and every 8 h thereafter. After discharge, patients were medicated with meloxicam^9^ (0.1 mg/kg, VO) every 24 h for 4 days and tramadol chlorhydrate (3 mg/kg, VO) every 8 h for 5 days.

### Evaluation of surgical stress

Patients were evaluated in 10 different times, including preoperative, transoperative, immediate, and late postoperative periods. The first time was at the initial examination when the tumor was diagnosed (T1). This happened approximately 10 day before surgery in a consulting room, in the presence of the owner. During the preoperative period, patients were evaluated at three time points, as follows: T2, immediately before premedication; T3, 15 min after premedication; T4, five min after induction and orotracheal intubation. During the transoperative period (T5), patients were evaluated every 5th min, and their physiologic parameters were kept stable. During the immediate postoperative period (first 24 h), while they were still using compression bandages, patients were evaluated at three time which were: T6, 2 h after surgery; T7, 12 h after surgery; T8, 24 h after surgery. During the late postoperative period, animals were evaluated at two time points, according to scheduled appointments: T9, 48 h after surgery, when compression bandages were removed and surgical wound was evaluated; and T10, 10 day after surgery, when stiches were removed.

Blood samples were drawn through jugular venipuncture at T2, T4, T6, and T8 to measure objective serum markers, invariably in the afternoon. Serum cortisol level was assessed through chemiluminescence after blood storage in sterile tubes with clot activator, for as long as 24 h. A reference range of 1.0–6.0 mcg/dL was used [9]. Glycemia was assessed, immediately after collection of whole blood, in a portable Trackease Smart System (Trackease stripes)^10^. A reference range of 70–110 mg/dL was used [9]. Samples from T2 (before administration of any drugs) and T8 (24 h after surgery) also were stored under refrigeration in sterile tubes with EDTA (ethylenediamine tetraacetic acid) for as long as 4 h for hematological assessment. Complete blood counts (CBC) were performed with Abacus Veterinary Hematology Analyzer^11^. Differential leukocyte count and morphologic evaluation of cells were performed in blood smears stained with panoptic^12^ under optic microscopy. Reference values in accordance with Jain [10] were used.

Physiological parameters evaluated included heart rate, respiratory rate, body temperature, and arterial pressure. The first three parameters were assessed in all 10 evaluation points. Reference ranges of 60–160 beats per minute, 18–36 breaths per minute, and 37.5–39.2 °C, respectively were used [11]. Non-invasive arterial pressure was assessed through oscillometric method with a Veterinary Digital Blood Pressure Device^12^ and a cuff with a width of 40 % limb circumference, positioned on the middle third of the right radius. Arterial pressure was evaluated on T1–T10 with patient on dorsal recumbency during surgical procedure or in left lateral recumbency on other evaluation times. Values for each patient, in each assessment time, were the result of an arithmetic average of three measurements. For systolic arterial pressure (SAP), diastolic arterial pressure (DAP) and mean arterial pressure (MAP) reference ranges of 80–120 mmHg, 60–80 mmHg and 65–96 mmHg, respectively were used [11]. When animals were in hospital cages during evaluation periods (T2, T6–T10), they were taken into a quiet room away from other animals for 5–10 min, so that they could adapt before measurements. During T5, hemoglobin oxygen saturation was also measured with a digital pulse oximeter.^11^

**Fig. 1:**
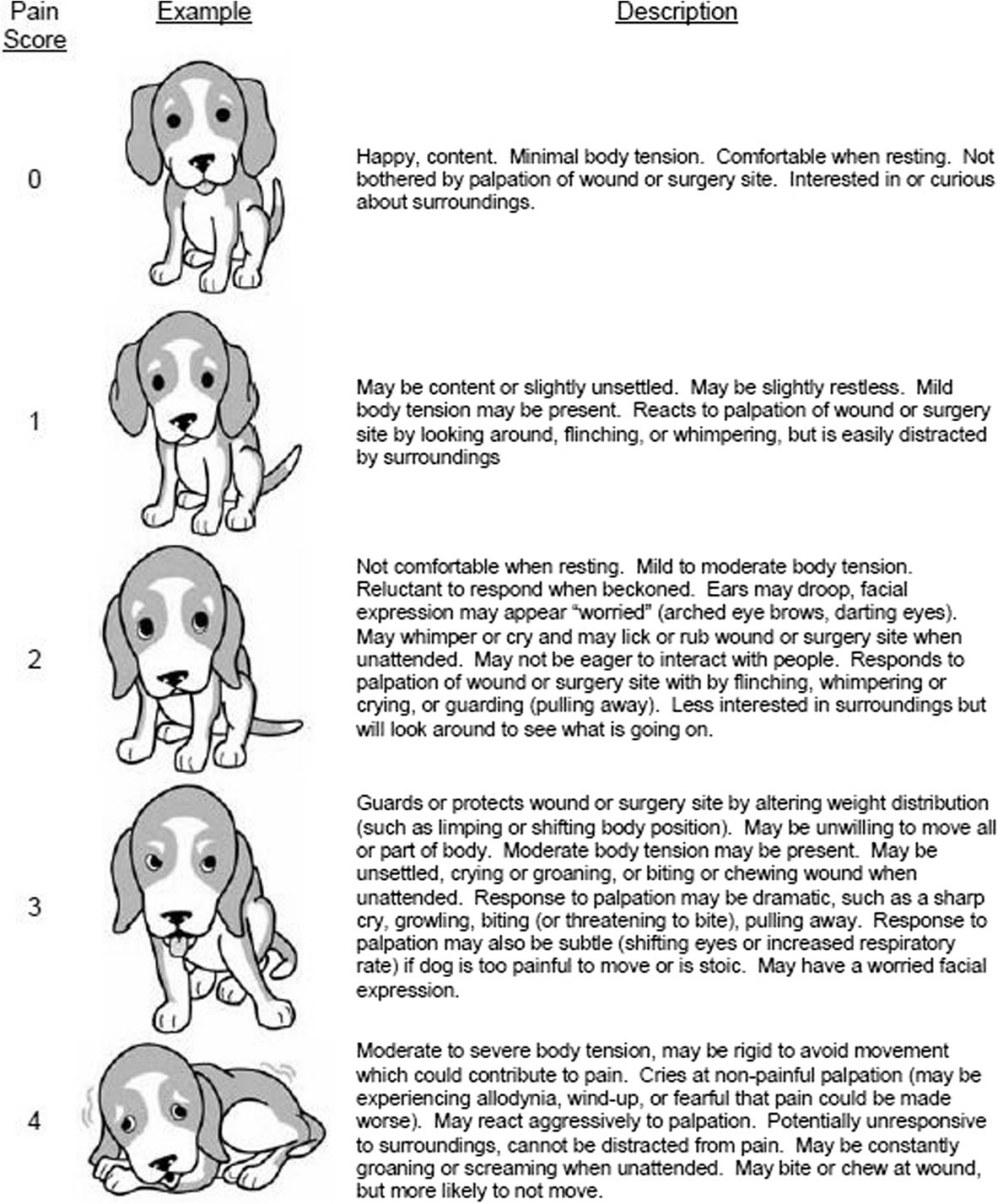
Postoperative nociceptive evaluation scale proposed by University of Colorado (adapted from Hellyer et al. [12])

For the postoperative nociception study (T6–T10), two nociception evaluation scales were used. The University of Colorado Scale, adapted from Hellyer et al. [12], considered behavior changes and palpation response to an area of 2 cm^2^ around surgical wound, with a score from 0 to 4 (Fig. 1). The Postoperative Nociception Evolution Scale was developed according to Malm et al. [13], and it considered five physiological nociception markers (objective) and five behavior markers (subjective), with a score from 0 to 26 (Table 1). For both scales, higher scores were associated with a greater nociception response. Because there was no intent to prove result repeatability, evaluations were performed by a single trained person, as described by Slingsby and Water-Pearson [14] for cats and by Maticic et al. [15], Moll et al. [16], and Kim et al. [17] for dogs.

**Table 1:** Postoperative nociceptive evolution scale (adapted from Malm et al. [13])

Patient:	Record number:	Date:	Time:
Category	Observer		Score
• Behavior data:			
Mental state/Temperament	Similar to state before surgery		0
	Changed, but submissive		1
	Changed but slightly friendly		1
	Changed, but distrustful		2
	Changed, but aggressive		3
Activity level/disposition	Similar to level before surgery/feeding		0
	Resting (in recumbency or standing) or sleeping		1
	Restless		2
	Rolling or self-mutilating		3
Posture	Recumbency, but rising when stimulated, seating, or standing with the head up		0
	Moving, seated, or standing with the head down		1
	Recumbency, but not rising when stimulated		2
	Protecting affected area (including fetal position)		2
	Standing in prayer or arched posture		3
Vocalization	Does not vocalize		0
	Slight vocalization when touched or changing position		1
	Exaggerated vocalization when touched or changing position		2
	Spontaneous vocalization, stops when touched		2
	Continuous spontaneous vocalization		3
Response to surgical wound area palpation (2 cm)	No alteration		0
	Slight discomfort, watchful to palpation, and looking at manipulated area, but relaxed abdominal wall		1
	Moderate discomfort, watchful to palpation, and looking at manipulated area, but rigid abdominal wall		2
	Immediate response with movement and attempt to evade painful stimulus		2
	Immediate response with vocalization, attempt to evade painful stimulus, may act aggressively		3
• Physiological data:			
Heart rate	0 to 20 % above preoperative value		0
	20 to 50 % above preoperative value		1
	50 to 100 % above preoperative value		2
	100 % above preoperative value		3
Respiratory rate	0 to 20 % above preoperative value		0
	20 to 50 % above preoperative value		1
	50 to 100 % above preoperative value		2
	100 % above preoperative value		3
Body temperature	Normal temperature		0
	Temperature below reference values		1
	Temperature above reference values		1
Pupils	Normal		0
	Dilated		2
Excessive drooling	Absent		0
	Present		2

0 to 26 score; the higher the score, the greater the postoperative pain

Rescue analgesia with intramuscular morphine injection in a dosage of 0.3 mg/kg was used in patients that reached twelve or more points on the Postoperative Nociception Evolution Scale.

### Evaluation of postoperative complications

According to protocols described in studies by Hedlund [7] and Al-Asadi et al. [18], nine postoperative complications were evaluated: posterior limb edema, hematoma, subcutaneous emphysema, dehiscence, surgical wound contamination, bleeding, seroma, hyperesthesia, and allodynia.

During the immediate postoperative period, patients were examined for these complications 2, 12, and 24 h after surgery, matching nociception evaluation times T6, T7, and T8, respectively. During late postoperative period, complications associated with surgical procedure were evaluated two days after surgery, in the 48 h follow-up appointment (T9) and 10 d after surgery, immediately before suture removal (T10).

Posterior limb edema, hematoma, subcutaneous emphysema and surgical wound dehiscence severity varied, so these four complications were classified as discrete, moderate, and intense. Posterior limb edema was classified as discrete when it did not cause mechanical complications to the patient; moderate when it interfered with patient ambulation; and intense when it prevented patient ambulation. Hematoma and subcutaneous emphysema were classified as discrete when restricted to one spot in a 2-cm^2^ area around surgical wound; moderate when they occurred in more than one spot in a 2-cm^2^ area around surgical wound; and intense when they exceeded a 2-cm^2^ area around surgical wound. Dehiscence was classified as discrete, moderate, and intense when it occurred in < 10 %; ≥ 10 to < 30; and ≥30 % of the surgical wound, respectively.

Other postoperative complications were accessed without an intensity classification. Wound contamination was positive when purulent discharge was seen in the surgical wound. Seroma was positive when there was accumulation of serous liquid in the surgical wound. Peripheral and central sensitivity responses were distinguished according to type of stimuli triggered. Hyperesthesia was evaluated applying a gentle pressure in the middle of a 2-cm² area around surgical wound. Allodynia was evaluated applying the same gentle pressure in the middle of a distant area (between 5 and 6 cm) from surgical wound.

### Experiment design and statistical analyses

A subdivided parcel design was conducted. Surgical procedures consisted of parcels, as regional mastectomy was named Group 1 and unilateral radical mastectomy was named Group 2. Evaluation periods were subparcels, and each animal was considered an experiment unit or repetition. Differences were considered statistically significant when *P* < 0.05.

Before comparison tests were used, parametric data was tested for normalcy (Kolmogorov-Smirnov test) and homoscedasticity. Parametric data, with normal distribution of probabilities and variance homogeneity, were reported as mean and standard error, and subjected to analyses of variance, with Fisher’s test to compare parcels and Tukey test to compare subparcels. Non-parametric data and values that did not meet normalcy and homoscedasticity criteria were reported as median and subjected to Mann–Whitney test to compare parcels and to Kruskal-Wallis (one-way analysis of variance), followed by Dunn’s (non-parametric pairwise multiple comparisons procedure) post-hoc to compare subparcels. Behavior aspects identified after T6 (without anesthetic drugs) resulted in 144 observations (72 for each parcel). Frequency dispersion according to Postoperative Nociception Evolution Scale grading was analyzed with chi-squared test. CBC observations and postoperative complications were similarly analyzed. Patients with mammary tumors presenting with adherences or ulceration were compared with patients who underwent the same surgical technique, but without these characteristics, using the same statistics model. To test and assess association between studied variables in each parcel (Groups 1 and 2), Pearson correlations were used for parametric data and Spearman correlations were used for non-parametric data. Significant correlations were considered strong when they were present in over 49 % of the studied population (*r* > 0.07), moderate when they were present in 9 to 49 % (0.3 < *r* < 0.07) and weak when they were present in less than 9 % of the population (*r* < 0.3).

## Results

Group 1 patients were between 5 and 13 years old (9.8 ± 2.4) and had a mean body weight of 9.6 kg. Group 2 patients were 6–14 years old (10.5 ± 1.9) and had a mean body weight of 11.3 kg. Statistical significance for each variable is represented in Table 2.

**Table 2:** Statistical significance for different postoperative parameters between regional (Group 1) and radical (Group 2) mastectomy

Parameter	Regional mastectomy	Radical mastectomy	Statistical significance
Length of procedure	35 ± 11 min	48 ± 16 min	*P* < 0.02
Wound surgical size	11 ± 4 cm	24 ± 7 cm	*P* < 0.0001
Serum cortisol in T6	4.2 ± 2.3 mcg/dL	8.2 ± 5.3 mcg/dL	*P* < 0.004
Glycemia in T6	145,7 ± 46,0 mg/dL	192,6 ± 61,1 mg/dL	*P* < 0.01
Glycemia in T8	108,8 ± 22,2 mg/dL	121,8 ± 25,7 mg/dL	*P* < 0.04
Systolic arterial pressure in T6	91,0 ± 15,7 mmHg	102,9 ± 24,4 mmHg	*P* < 0.03
Postural disorders	88.9 %	98.5 %	*P* < 0.02
Response to surgical wound palpation	83.3 %	97.2 %	*P* < 0.05
Score in University of Colorado Scale (median)	T6 (2), T7 (1), T8 (1), T9 (1)	T6 (3), T7 (2), T8 (2), T9 (2)	*P* < 0.05
Score in Postoperative Nociception Evolution Scale (median)	T6 (6,5), T7 (4,5), T8 (4,0) T9 (4,0), T10 (3,0)	T6 (11,5), T7 (9,0), T8 (8,5), T9 (6,5), T10 (5,0)	*P* < 0.05
Rescue analgesia	1/18 patients	10/18 patients	*P* < 0.005

T6, 2 h after surgery; T7, 12 h after surgery; T8, 24 h after surgery; T9, 48 h after surgery; T10, 10 days after surgery

Considering all surgical procedures performed in this study (*n* = 36), duration of surgery varied from 20 to 90 min (42 ± 14 min). Animals that underwent regional mastectomy had a shorter procedure duration and a smaller surgical wound (35 ± 11 min and 11 ± 4 cm) than those that underwent radical mastectomy (48 ± 16 min and 24 ± 7 cm), with *P* < 0.02 and *P* < 0.0001, respectively. Correlation between these two variables was positive and moderate (*P* < 0.0001; r_P_ = 0.593).

Non-invasive arterial blood pressure evidenced difference in SAP values during T6, with higher values in animals subjected to radical mastectomy (*P* < 0.03).

**Fig. 2:**
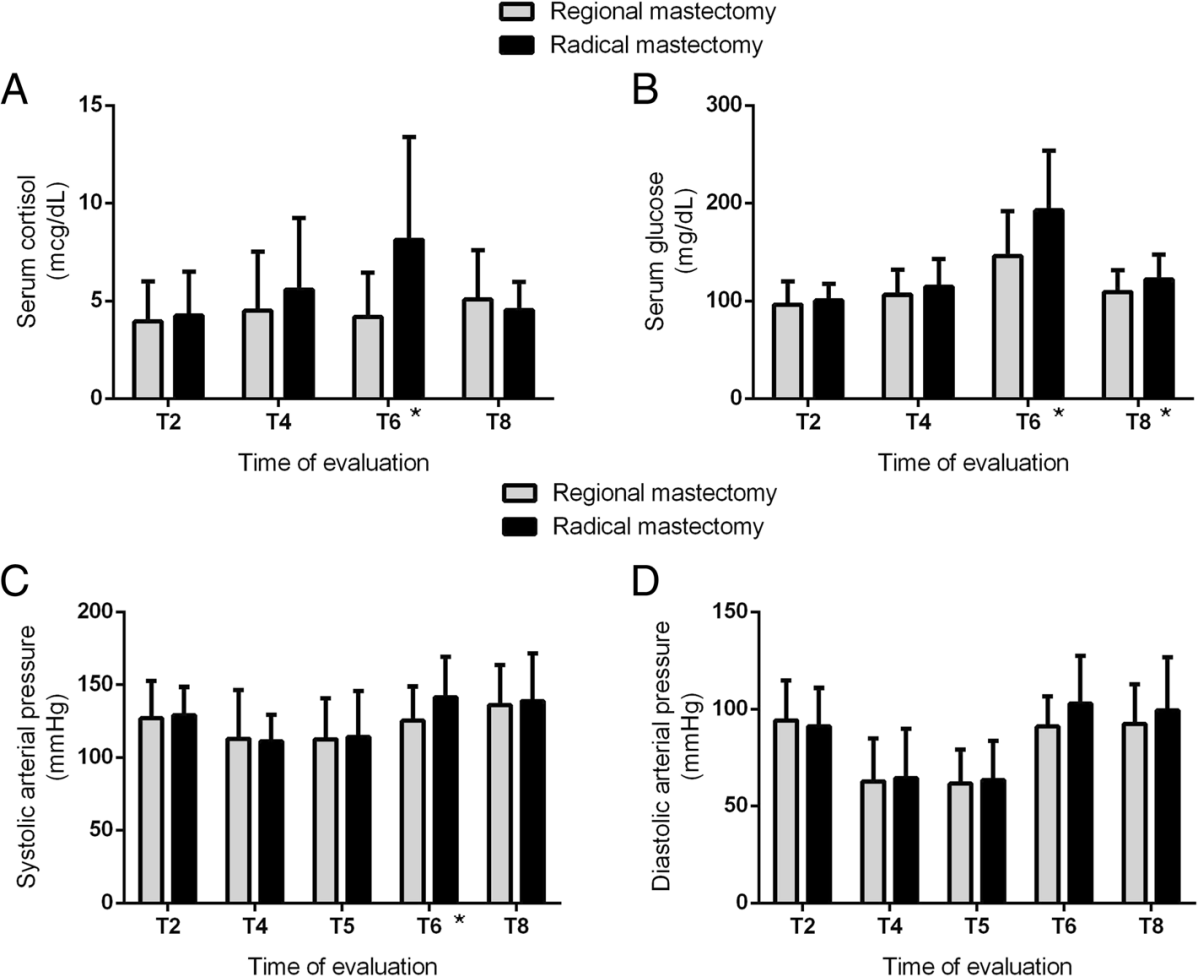
Graphic representation with mean values and standard deviation for serum cortisol (**a**), serum glucose (**b**), systolic arterial pressure (**c**), and diastolic arterial pressure (**d**) at T2 (immediately before premedication), T4 (5 min after general anesthesia and orotracheal intubation), T5 (immediately after orotracheal intubation), T6 (2 h after surgery), and T8 (24 h after surgery) in dogs undergoing regional (*n* = 18) and radical (*n* = 18) mastectomy. Significant differences for serum cortisol (Mann–Whitney, *P* < 0.004), on T6; serum glucose, on T6 (Fisher, *P* < 0.01) and T8 (Fisher, *P* < 0.04); and systolic arterial pressure, on T6 (Fisher, *P* < 0.03), are marked with stars

Mean concentrations of serum cortisol in each evaluation for both groups are represented in Fig. 2. Difference between two groups was evidenced in T6, with lower levels in animals that underwent regional mastectomy (4.2 ± 2.3 versus 8.2 ± 5.3 mcg/dl), with *P* < 0.004 (Mann–Whitney). Concerning glycemia, animals subjected to radical mastectomy had higher glycemic values in T6 (Fisher, *P* < 0.01) and T8 (Fisher, *P* < 0.04). The results for those parameters in T6 were not significantly correlated with surgery duration, but with surgical wound extension (*P* < 0.003, r_S_ = 0.486 for serum cortisol, and *P* < 0.02, r_S_ = 0.380 for glycemia).

Considering the number of patients with hematological abnormalities, there was a significant increase in leukocyte (*P* < 0.03) and neutrophil counts (*P* < 0.0002) for both groups in the postoperative period. A significant increase (*P* < 0.007) in neutrophil count, accompanied by right shift (significant presence of hypersegmented neutrophils) was identified after surgery only in the group that underwent radical mastectomy.

During the postoperative period (144 posture observations) alterations were seen in 88.9 % of Group 1 observations (83.3 % moving, sitting, or standing with head facing down and 5.6 % in recumbency not responding to stimulus, protecting affected area) and 98.5 % of Group 2 observations (81.9 and 16.7 %, respectively). Animals that underwent radical mastectomy had greater postural disorders (*P* < 0.02). Responses to surgical wound palpation were seen in 83.3 % of Group 1 observations (56.9 % with slight discomfort and relaxed abdominal wall and 26.4 % with moderate discomfort, tense abdominal wall, or immediate response with an attempt to evade painful stimulus). Responses to surgical wound palpation were seen in 97.2 % of Group 2 observations (29.2 % with slight discomfort and relaxed abdominal wall; 61.1 % with moderate discomfort, tense abdominal wall, or immediate response with attempt to evade painful stimulus and 6.9 % with immediate reaction, vocalization, and attempt to evade painful stimulus, and indications that aggressiveness might be present). More intense responses to surgical wound palpation were seen in animals that underwent radical mastectomy (*P* < 0.005).

Patients that underwent radical mastectomy achieved higher scores on the University of Colorado Scale in T6–T9 and T6–T10 in Postoperative Nociception Evolution Scale (*P* < 0.05). During 180 postoperative evaluations, rescue analgesia was necessary for one patient (only in one T6 evaluation) for the group that underwent regional mastectomy, but for 10 patients (in 13 evaluations, of which nine were in T6, two in T7 and two in T8) for the group that underwent radical mastectomy (*P* < 0.005).

Postoperative complications were diagnosed in 14 patients of each group (77.8 %), regardless of severity. The number of postoperative complications diagnosed in each evaluation period according to surgical technique is specified in Table 3. Postoperative complications were seen in 57.8 and 91.7 % of evaluations for Groups 1 and 2, respectively, in 180 evaluations (T6–T10). Therefore, greater postoperative complications were observed in dogs that underwent radical mastectomy (*P* < 0.03). Posterior limb edema was considered discrete in all observations. Resolution occurred in three to seven days after treatment with hot dressings and moderate exercise. Natural absorption and complete resolution of hematomas also were seen in all cases within 3 to 8 days. Surgical wound dehiscence was considered moderate in 80 % (4/5) of Group 2 observations, while it was considered discrete in the remaining observations. Secondary intention healing was observed in all patients within 7 to 14 days, with usage of saline lavage and sunflower oil bandages. Hematomas were discrete, but were considered moderate in 37.5 % (9/24) of Group 1 observations, severe and moderate in 15.8 % (3/19), and in 47.4 % (9/19) of Group 2 observations, respectively. Only three animals presented discrete seroma in the inguinal region, with spontaneous resolution within 3 to 4 d. Subcutaneous emphysema was mostly discrete, except for one Group 2 observation, but natural absorption and complete resolution occurred in all cases within 3 to 5 days. Hyperesthesia and allodynia were rare in this study. Prescribed analgesics and rescue analgesia were sufficient to treat those sensitization reactions.

**Table 3:** Number of patients with postoperative complications in different times after regional (*n* = 18) and radical (*n* = 18) mastectomy

Postoperative complications	Group 1 Regional mastectomy						Group 2 Radical mastectomy					
	T6	T7	T8	T9	T10	Total	T6	T7	T8	T9	T10	Total
Posterior limb edema	0	1	1	4	4	10	0	4	4	4	2	14
Hematoma	0	0	0	12	12	24	0	0	0	10	9	19
Dehiscence	0	0	0	1	2	3	0	0	0	1	4	5
Subcutaneous emphysema	0	0	1	1	0	2	2	0	0	1	1	4
Infection	0	0	0	5	4	9	0	0	0	6	9	15
Bleeding	0	0	0	1	0	1	0	0	1	0	0	1
Seroma	0	0	0	1	1	2	0	0	0	2	0	2
Hyperesthesia	1	0	0	0	0	1	0	1	1	1	0	3
Allodynia	0	0	0	0	0	0	1	1	1	0	0	3
Total	1	1	2	25	23	52	3	6	7	25	25	66

T6, 2 h after surgery; T7, 12 h after surgery; T8, 24 h after surgery; T9, 48 h after surgery; T10, 10 days after surgery

## Discussion

Al-Asadi et al. [18] reported a mean duration of 50 min for surgical removal of mammary tumors, but they did not differentiate among surgical techniques used. In this study, animals that underwent regional mastectomy had shorter procedure duration and smaller surgical wound.

In the present study, no significant changes in serum levels of glucose and cortisol were seen immediately after anesthetic induction (T4), indicating that animal manipulation, including physical restraint, venipuncture, drug administration, and orotracheal intubation did not interfere with neuroendocrine and metabolic functions, in accordance with observations of Church et al. [19] and Caldeira et al. [20]. Although Yoder and Wolf [21] reported association between duration of surgery and cortisol levels in postoperative period of dogs that underwent nephrectomy, there was no correlation, in this study, between those parameters, nor did we find an association between duration of surgery and glycemia. Nevertheless, cortisol and glycemia elevation at T6 were moderately correlated with surgical wound extension, probably related to greater nociceptive effect triggered by radical mastectomy. Peripheral and central nociceptive stimulation can increase release of cortisol [22–24]. A hyperglycemic state also may be established in painful situations owing to catabolic effect of cortisol and peripheral resistance to insulin, as described by Cunningham [25]. Decrease in these levels in the subsequent evaluation period (T8) indicates a reduction in central sensitization and a decrease in inflammatory reaction, with reduced influence in neuroendocrine and metabolic responses, as noted by Fox et al. [26].

Higher values of SAP in animals undergoing radical mastectomy is probably secondary to sympathetic nervous system activation and shows a larger nociceptive response in animals undergoing radical mastectomy [24, 27, 28].

In this study, a significant increase in neutrophil counts was seen after surgery in the group that underwent radical mastectomy, although a greater number of dogs showed neutrophilia after surgery in both groups, as previously described [22, 29, 30]. Hypersegmentation of neutrophils (right neutrophil shift) identified in post-operative samples for Group 2 is a result of increased neutrophil nucleus maturation. This is probably related to increased level of serum steroid, a condition that prolongs circulating time and decreases neutrophil migration [30, 31]. This was not a common finding for Group 1, which experienced less surgical trauma, followed by reduced stress response.

General behavior disorders in dogs may be associated with painful experiences and include temperament modifications, activity level, posture, enhanced abdominal muscle tonus, and attempt to protect the affected area [13, 32–35]. In this study, these disorders were more frequent in patients that underwent radical mastectomy. Arched or praying posture (score 3) was not identified in any of the observations, probably due to rescue analgesia. According to Hardie [35], extremely painful experiences might be necessary for expression of obvious nociceptive behaviors. As demonstrated in this study, surgical wound palpation might elicit different reactions depending on individual features, intensity of pain, discomfort or stress [13, 24, 32, 35, 36]. Severe reactions with attempts to bite the observer were seen only in animals that underwent radical mastectomy and suggest greater postoperative pain [13, 24, 35].

The University of Colorado Scale showed evolution of nociception or discomfort in animal postoperative period, without considering physiological parameters, and evaluating only behavior features. The University of Colorado Scale is simple to administer but not very sensitive, given that it has only four categories. In contrast, Postoperative Nociception Evolution Scale is the result of behavior and physiological (heart rate, respiratory rate, body temperature, pupil dilation and salivation) parameters.

Rescue analgesia was used for patients that achieved 12 points—representing a score below 50 % of the scale—in the Postoperative Nociception Evolution Scale. This was done to avoid underestimating patient pain, because the decision to use rescue analgesia was based on a subjective method that has not been validated [37]. Postoperative pain was more easily controlled in Group 1 patients. This suggests that continuous infusion of low-dose analgesic combinations, such as fentanyl-lidocaine-ketamine or morphine-lidocaine-ketamine, during inhalant anesthesia and after surgery should be considered in animals undergoing radical mastectomy to improve trans- and post-operative analgesia [38].

Behavior signs observed in both scales may be related to patient emotional disorders, such as anxiety, fear and excitement [13]. Similarly, physiological parameters may also be influenced by patient emotional status and drugs administered [13, 36]. A specific marker to evaluate nociception and pain in animals does not yet exist; however, association of several objective and subjective markers could be the most reliable method for evaluating pain [24, 36].

Post-operative complications were relatively common in dogs that underwent mastectomy in this study but were more frequent in the radical approach. Posterior limb edema was mainly related to inguinal lymph node removal causing inguinal drainage impairment [7, 18]. Subcutaneous emphysema was related to removal of mammary tissue adhered to subcutaneous tissue [7]. According to Hedlund [7], canine mammary glands have an extensive blood supply and a complex mammary tissue lymphatic drainage that might favor development of hematomas and seromas. In this study, the choice not to use electrocautery and the usage of compressive bandages for 48 h after surgery may be related to reduced incidence of seroma [7, 39].

There was no intent, in this study, to report long-term results. Previous data about disease control and overall survival in dogs with mammary tumors are conflicting, but few studies have evaluated surgical stress triggered by different techniques. Stratmann et al. [5] found a greater probability of new ipsilateral tumor development in noncastrated bitches undergoing regional mastectomy. Although a correlation was found between histological types, a detailed histological classification was not performed. As demonstrated by MacEwen et al. [3], a recent study advocated less aggressive surgical procedures according to clinical features such as staging, number and size of lesions, presence of adherences, and tumor site. Dogs in this study were not spayed and there was no difference in new tumor development, disease-free interval, or overall survival [40]. Prophylactic surgery by means of radical mastectomy obviously can prevent new mammary neoplasm development in the future [8]. However, it is an invasive procedure that might result in surgical stress and postoperative complications, as demonstrated in this study. In addition, it is not proven yet that dogs with history of malignant mammary tumor have greater risk of developing new mammary neoplasms [8, 40]. Empiric risk may be reduced by regular re-examination and prompt intervention if a new mass is found [40].

## Conclusions

Radical mastectomy resulted in longer surgical duration, greater nociceptive stimulus, surgical stress, and higher incidence of postoperative complications when compared with regional mastectomy in dogs. Although it was not the aim of this study to evaluate long-term results, canine mammary tumor surgical treatment should be based on lymphatic drainage, number and size of lesions, and established prognostic factors. Disregard of these criteria may result in potentially unnecessary aggressive surgical procedures. Each patient should be individually assessed and directed for each surgical technique, taking into account the postoperative recovery and patient quality of life.

## Footnotes

1 Cefariston®, Ariston, Morón, Buenos Aires, Argentina.

2 Cefalexina, Medley, Campinas, São Paulo, Brazil.

3 Acepran®, Vetnil, Louveira, São Paulo, Brazil.

4 Dimorf®, Cristália, São Paulo, São Paulo, Brazil

5 Fresofol 1 %®, Fresenius Kabi Brasil Ltda, Campinas, São Paulo, Brazil

6 Isothane® 100 ml, Baxter Hospitalar Ltda., Santo Amaro, São Paulo, Brazil.

7 Maxicam®, Ourofino Agronegócio, Cravinhos, São Paulo, Brazil.

8 Cloridrato de Tramadol, União Química Farmacêutica Nacional S/A, Jabaquara, São Paulo, Brazil.

9 Meloxivet®, Duprat, Rio de Janeiro, Rio de Janeiro, Brazil.

10 Trackease Smart System – Accumed, Duque de Caxias, Rio de Janeiro, Brazil.

11 Abacus Veterinary Hematology Analyzer, Diatron, São Paulo, São Paulo, Brazil.

12 Veterinary Digital Blood Pressure Device, Contec Medical Systems Co., Qinhuangdao, China.
